# Unpacking Mentorship: A Review of Its Role in Contemporary Postgraduate Surgical Education

**DOI:** 10.7759/cureus.99455

**Published:** 2025-12-17

**Authors:** Daniel Thomas, Thomas Oldfield, Hannah Bamford

**Affiliations:** 1 Urology, Royal Hallamshire Hospital, Sheffield, GBR; 2 Surgery, Mid Yorkshire Teaching NHS Trust, Wakefield, GBR; 3 Plastic Surgery, Mid Yorkshire Teaching NHS Trust, Wakefield, GBR

**Keywords:** ent surgeon, health professional education, mentor-mentee program, mentorship program, obstetrics & gynaecology, plastic surgeons, resident surgeon, speciality registrar, surgery general, surgical careers

## Abstract

Mentorship is a cornerstone of postgraduate surgical education, supporting the development of clinical competence, research capability, professional identity, and psychological well-being. Despite its recognised importance, access to effective mentorship during surgical training remains inconsistent. This review aimed to evaluate the role, impact, and challenges of mentorship within postgraduate surgical education. A systematic review was conducted according to the Preferred Reporting Items for Systematic Reviews and Meta-Analyses guidelines. Comprehensive searches of Ovid Medline, Embase, and Emcare identified studies published between 2015 and 2025 that examined mentorship in surgical residency programs. Eligible studies explored mentorship models, outcomes, and implementation strategies. Data were extracted on study design, participant characteristics, mentorship approaches, and key outcomes. Due to methodological heterogeneity, findings were synthesised narratively. In total, 13 studies met the inclusion criteria, encompassing a range of surgical specialties and mentorship formats. Mentorship demonstrated consistent benefits across multiple domains. Structured programs enhanced academic productivity, with mentored residents producing more publications, engaging in research, and achieving greater scholarly output. Mentorship also improved technical skills, procedural confidence, and reflective practice. Professionally, mentored trainees reported stronger leadership development, clearer career trajectories, and higher examination success. Psychosocially, mentorship was associated with reduced burnout, improved confidence, and greater work-life balance. Peer and near-peer mentoring models further strengthened collegiality and accessibility. However, barriers such as limited time, inadequate mentor training, and inconsistent institutional support were frequently reported. Mentorship is integral to postgraduate surgical training, offering substantial academic, clinical, and professional benefits. Structured, well-resourced programs with protected time, trained mentors, and formal institutional recognition yield the most sustainable outcomes. Future initiatives should focus on standardised evaluation frameworks, long-term impact assessment, and innovative mentorship delivery models. Embedding mentorship as a core component of surgical education is essential for cultivating competent, resilient, and future-ready surgical professionals.

## Introduction and background

Mentorship is a cornerstone of professional and personal development, particularly in healthcare. It represents a relationship between a mentor and mentee that facilitates learning and professional growth in domains such as skill acquisition, knowledge, and attitude [1]. Beyond technical instruction, mentorship is a dynamic, enduring process rooted in guidance, support, and reflective practice. Over the past two decades, it has become increasingly recognised as central to professional identity formation, with evidence highlighting its benefits for both mentors and mentees [2].

Historically, mentorship in medicine emerged from the apprenticeship model, where learners developed competence through close observation of experienced practitioners. Today, mentorship has evolved into structured, formalised relationships promoting holistic development, encompassing clinical proficiency, research productivity, career advancement, and psychological well-being [3]. This shift reflects the recognition that effective mentorship enhances learning outcomes, builds resilience, and nurtures a positive educational culture within healthcare organisations.

At the undergraduate level, mentorship’s impact is well established. Studies show that it improves academic performance, research engagement, and professional identity formation among medical students [4,5]. Students involved in mentorship relationships report greater satisfaction with training, clearer career trajectories, and stronger motivation for postgraduate education. Mentorship also influences specialty selection, with mentees frequently pursuing the disciplines of their mentors [6]. These findings demonstrate that mentorship not only supports immediate learning objectives but also shapes long-term professional pathways.

The benefits of mentorship extend to mentors themselves. Many describe mentorship as personally fulfilling and professionally enriching, providing opportunities for reflection, teaching skill enhancement, and leadership development [7]. Mentorship thus functions as a reciprocal relationship that strengthens educational culture and contributes to workforce sustainability.

Despite these advantages, mentorship within postgraduate surgical education remains inconsistent [8]. Surgical training is uniquely demanding, requiring mastery of complex technical skills alongside judgment, resilience, and teamwork. Transitioning from medical school to postgraduate surgical training introduces steep learning curves, high workloads, and psychosocial challenges. In this environment, mentorship can offer essential guidance, promoting reflective practice and supporting trainee well-being. Yet, studies indicate that mentorship opportunities in surgery are often limited, fragmented, or informal [9].

A 2019 survey of U.S. general surgery residents found that more than one-third reported inadequate mentorship [8]. Even where formal programs exist, barriers such as demanding clinical schedules, lack of protected time, poor institutional support, and mismatched mentor-mentee pairings undermine success [10]. These challenges reveal a persistent gap between the recognised value of mentorship and its practical implementation.

This gap is concerning because mentorship significantly influences surgical career choice. Positive mentorship during medical school increases the likelihood of students pursuing surgery [6]. The lack of structured mentorship at the postgraduate level represents a missed opportunity to reinforce professional identity, support development, and mitigate attrition in a high-pressure specialty. Without effective mentorship, trainees may experience isolation, burnout, and uncertainty about career progression, potentially affecting both their well-being and patient care outcomes [11].

Professional organisations have acknowledged these issues and begun promoting structured mentorship frameworks. The Royal College of Surgeons of England, for instance, launched an online mentorship platform in 2025 to embed mentorship as a core surgical competency. Such initiatives reframe mentorship as integral to surgical education and professional practice rather than as an optional supplement. Early feedback from residents suggests strong demand for structured mentorship and clearer guidance throughout training [12].

However, the mere establishment of mentorship programs does not guarantee success. Effective mentorship requires institutional and cultural support, including protected time for mentor-mentee interactions, appropriate mentor training, and defined expectations for both parties [13]. Programs should also reflect the unique demands of surgical training, balancing operative exposure, service commitments, and educational goals. Embedding mentorship into formal curricula ensures equity of access and consistency across institutions.

Current literature affirms that structured mentorship has measurable benefits for surgical trainees, including improved operative confidence, accelerated skill development, enhanced academic output, and greater career satisfaction [14]. Mentorship also fosters diversity and inclusion by supporting underrepresented groups and promoting equitable access to advancement. Moreover, effective mentorship cultivates leadership, professionalism, and lifelong learning, competencies essential to future surgical educators and leaders [15].

Despite compelling evidence, research on mentorship in surgical residency remains limited and heterogeneous. Few studies examine long-term outcomes such as promotion to consultant level, sustained academic productivity, or leadership attainment. Additionally, the characteristics of successful mentorship relationships-mentor qualities, matching processes, and institutional framework are underexplored.

Accordingly, this review aims to evaluate and synthesise existing evidence on the role and impact of mentorship in postgraduate surgical education within recent years; therefore, a time frame of 10 years was chosen to simulate changing surgical practice. It aims to identify key domains of influence, namely, trainee well-being, professional development, academic productivity, and career progression, while highlighting existing gaps and opportunities. By critically analysing current evidence, this review seeks to inform the development of effective, sustainable mentorship models and advocate for institutional investment in structured mentorship frameworks supported by protected time and adequate resources.

## Review

Methodology

**Figure 1:**
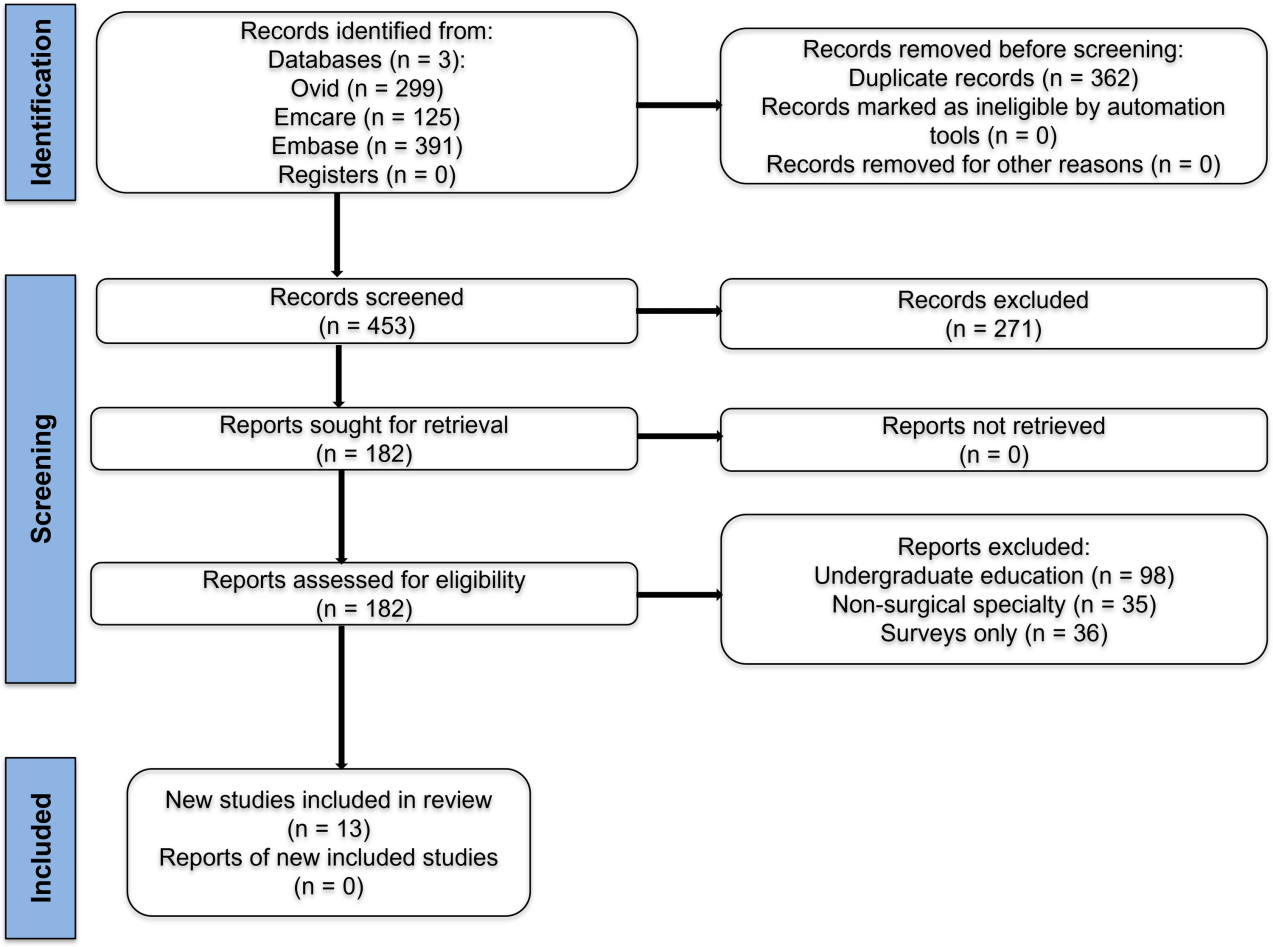
Preferred Reporting Items for Systematic Reviews and Meta-Analyses (PRISMA) flowchart representing study selection process.

A systematic literature review was undertaken to examine the role of mentorship in postgraduate surgical education. The review was conducted according to the Preferred Reporting Items for Systematic Reviews and Meta-Analyses (PRISMA) guidelines [16], as shown in Figure 1.

Search Strategy

A comprehensive search of Ovid Medline, Embase, and Emcare databases was performed to identify relevant studies published between January 2015 and July 2025. Both Medical Subject Headings (MeSH) and free-text terms in the title and abstract were used to capture variations in terminology related to surgery, surgical trainees, and mentorship.

The Ovid Medline, Embase, and Emcare search strategy was as follows: (Surgical or Surgery or surgeon) or (Internship and Residency/ or Surgeons/ or Surgical trainee.) and (trainee or registrar or resident or junior) and (mentor* or Coach* or mentorship or Mentors/) and (“2015 - 2025”). This strategy yielded 815 citations. After removing duplicates, titles and abstracts were screened independently by two reviewers. Full texts were retrieved for studies that met the inclusion criteria or where eligibility was uncertain. Reference lists of included articles were hand-searched to identify additional relevant studies.

Inclusion and Exclusion Criteria

Studies were eligible if they examined mentorship within postgraduate or residency-level surgical education. Only English-language articles published between 2015 and 2025 were included. Studies focusing solely on undergraduate education, non-surgical specialties, and opinion pieces were excluded.

Study Selection and Data Extraction

Two reviewers independently screened titles, abstracts, and full texts. Disagreements were resolved by consensus or consultation with a third reviewer. Data extracted included study design, participant characteristics, mentorship models, outcomes assessed, and key findings.

Quality Assessment and Data Synthesis

Methodological quality and risk of bias were assessed using the Joanna Briggs Institute (JBI) Critical Appraisal Checklists, selected according to study design. The included literature comprised observational cohort studies, qualitative studies, and mixed-methods evaluations; no randomised controlled trials (RCTs) were identified. Each study was appraised independently by two reviewers using the relevant JBI instrument, with attention to selection processes, measurement validity, confounding factors, and reporting transparency. Discrepancies were resolved by discussion or consultation with a third reviewer.

The majority of included studies demonstrated acceptable methodological rigour; however, limitations were noted in areas such as sampling representativeness, reliance on self-reported outcomes, short follow-up periods, and absence of comparator groups. Risk of bias was therefore judged to be moderate overall, reflecting the predominance of non-randomised observational designs and context-specific program evaluations. These methodological considerations informed the interpretation of the findings and contributed to the decision to adopt a narrative synthesis rather than meta-analysis.

Due to study heterogeneity in design, outcome measures, mentorship models, and reporting strategies, a descriptive narrative synthesis was conducted. Findings were organised thematically and supported, where appropriate, by descriptive numerical data extracted from the primary studies.

Results

A total of 13 studies were included in this review, each examining the role of mentorship in postgraduate surgical education across diverse specialties and contexts (Tables 1-4). The findings demonstrate that mentorship contributes significantly to residents’ academic productivity, clinical competence, professional identity, and psychosocial well-being. However, challenges such as time constraints, inadequate mentor training, and variable institutional support persist.

**Table 1:** Positive impacts of mentorship in postgraduate surgical education.

Illustrative studies	Theme	Description	Representative findings
Torres et al. [17]; Schroll et al. [18]; Coyan et al. [19]; Baig et al. [20]	Academic and Research Development	Mentorship enhances research engagement, scholarly productivity, and involvement in quality improvement initiatives	Residents in mentored programs produced more publications, presented at conferences, and demonstrated greater participation in academic activities
Schmalbach et al. [21]; Aho et al. [22]; Rajendran et al. [23]	Clinical Competence and Skill Development	Mentorship supports technical skill acquisition and reflective learning within surgical training	Structured mentorship improved procedural confidence, self-assessment, and examination performance among mentees
Haas et al. [24]; Ratan et al. [25]; Salles et al. [26]	Professional Identity and Leadership Formation	Mentorship aids trainees in defining their professional roles, leadership capacity, and long-term career goals	Mentees described mentors as role models who shaped their identity as surgeons and guided career choices
Schroll et al. [18]; Rajendran et al. [23]; Shah et al. [27]	Psychosocial Support and Well-being	Mentorship fosters resilience, motivation, and work-life balance by providing emotional and professional support	Mentored residents reported greater confidence, reduced stress, and improved sense of belonging within their programs
Rajendran et al. [23]; Ratan et al. [25]	Peer and Near-Peer Mentorship	Peer mentorship encourages collaborative learning and accessibility within surgical hierarchies	Junior–senior resident pairings promoted openness, feedback exchange, and collegial relationships

**Table 2:** Challenges and enablers of mentorship programs.

Studies	Theme	Challenges/Enablers	Representative findings	Implications for practice
Coyan et al. [19]; Schmalbach et al [21]; Shah et al. [27]; Khan et al. [28]	Time Constraints and Workload	Heavy clinical schedules limit the frequency and depth of mentor–mentee meetings	Both mentors and mentees cited a lack of protected time as the most significant barrier to sustained mentorship	Institutions should allocate protected time and formally recognise mentorship activities in workload planning
Rajendran et al. [23]; Salles et al. [26]; Shah et al. [27]	Mentor Availability and Training	Inadequate number of trained mentors and inconsistent mentor preparedness	Few programs provided formal mentor training; quality varied widely between departments	Develop faculty development programs to equip mentors with educational and interpersonal skills
Rajendran et al. [23]; Salles et al. [26]; Khan et al. [28]	Program Structure and Sustainability	Informal, ad hoc mentorship leads to variable engagement and unclear expectations	Structured programs with defined goals and regular meetings showed better participation and outcomes	Adopt formalised mentorship frameworks with scheduled reviews and institutional oversight
Schroll et al. [18]; Shah et al. [27]	Institutional and Cultural Support	Limited institutional recognition and a lack of funding reduce long-term sustainability	Programs with strong departmental support and recognition had higher retention and satisfaction rates	Embed mentorship within postgraduate curricula and institutional policy to ensure sustainability
Schroll et al. [18]; Aho et al. [22]; Rajendran et al. [23]; Ratan et al. [25]; Salles et al. [26]	Evaluation and Outcome Measurement	Lack of consistent evaluation tools and outcome indicators hampers program improvement	Most studies relied on self-reported satisfaction and lacked objective performance metrics	Develop standardised metrics to assess mentorship effectiveness across academic and professional domains

**Table 3:** Summary of key findings from identified studies on mentorship in postgraduate surgical education. QI = quality improvement; O&G = obstetrics & gynaecology; RRMT = resident research mentorship team

Study	Population	Mentorship strategies	Main outcomes	Limitations
Torres et al. [17]	24 (pre) and 27 (post) residents	Research committee provided guidance, feedback, and oversight	↑ Publications, ↑ journal impact factor, more fellowships pursued	Impact factor not the ideal measure; mandatory project change confounded results
Schroll et al. [18]	31 general surgery residents	Five QI mentor-led groups with regular meetings	↑ QI involvement, knowledge, and confidence	Impact of mentorship isolated from other program factors unclear
Coyan et al. [19]	34 cardiothoracic residents	Regular mentor meetings + statistical support	↑ Publications, presentations, textbook chapters	Time constraints; program length differences
Baig et al. [20]	99 resident research abstracts	Mentor publication record examined	Mentors with >25 publications → ↑ publication likelihood	Limited to association; mentor role unclear
Schmalbach et al. [21]	25 otolaryngology residents	Paired with seasoned reviewers for structured feedback	94% pass rate; high review quality; ongoing reviewer engagement	Mentor availability, admin demands, and time burden
Aho et al. [22]	12 general surgery residents	Three structured one-on-one mentoring sessions	Improved laparoscopic skills, self-directed learning	Variable mentoring content; possible observer effect
Rajendran et al. [23]	14 PGY1 and 14 PGY2 residents	Informal peer mentoring with flexible meetings	Improved confidence and goal setting	Inconsistent participation; scheduling conflicts
Haas et al. [24]	114 O&G residents (pre/post RRMT)	Four structured interdisciplinary meetings	↑ Publications, presentations, overall productivity	Protected time/funding may bias results; costly program
Ratan et al. [25]	5 faculty mentors, 15 junior faculty, 48 residents	Group mentorship for leadership workshops	Improved self-rated leadership score (non-significant)	Limited sessions; institutional context limits generalisability
Salles et al. [26]	76 general surgery residents	Junior–senior pairings with quarterly meetings	Generally positive feedback but poor participation	Low engagement; infrequent mentor-mentee meetings
Shah et al. [27]	73 studies (various surgical fields)	Not specified	Mentorship protective against burnout	Time constraints hinder implementation
Khan et al. [28]	Neurosurgeons from 80 US programs	Varied mentorship models (formal/informal)	Established programs (≥5 yrs) → ↑ board pass rates, output	Program director bias in survey responses
Mandel et al. [29]	30 plastic surgery graduates	Increased emphasis on mentorship post-transition	Academic track → ↑ research, academic careers	Self-selection bias; multifactorial program changes

**Table 4:** Synthesis of study conclusions. QIP = quality improvement plan; QI = quality improvement; SDL = self-directed learning; RRMT = resident research mentorship team

Studies	Conclusions
Torres et al. [17]	Research publications improved in quantity and quality after the implementation of a dedicated research program. This effect was not sustained after graduation on a per-resident basis, with the only sustained effect being a higher journal impact factor on a per-publication basis
Schroll et al. [18]	A structured QIP program increases resident involvement in QIPs, as well as their confidence and knowledge of QI processes and resources
Coyan et al. [19]	Implementation of a structured cardiac surgery research program facilitating mentoring significantly increased measures of academic productivity among residents
Baig et al. [20]	Resident projects are more likely to be published when they are supervised by faculty mentors with greater than 25 previous publications
Schmalbach et al. [21]	With appropriate mentorship, residents can perform scientific reviews at the level of their faculty counterparts
Aho et al. [22]	Mentor-guided SDL leads to increased practice which improved laparoscopic skills
Rajendran et al. [23]	Implementing a formal peer mentorship program has a positive impact for both mentor and mentees during the challenging transitional period of early surgical residency
Haas et al. [24]	The RRMT intervention was correlated with an increase in resident presentations and publications
Ratan et al. [25]	The program was positively received by all who participated; however, definitive leadership changes were not seen on resident self-assessment
Salles et al. [26]	The mentorship program was less commonly used than other aspects of the program, albeit still valued, with those who did meet with their mentor appreciating the guidance received
Shah et al. [27]	Structured mentorship programs are among the few evidence-based solutions for combating burnout
Khan et al. [28]	Programs with well-established mentorship had superior resident survey results, board pass rates, and scholarly activity
Mandel et al. [29]	The results of this study suggest mentorship is significant in career path selection and may encourage trainees to pursue careers in academia

Academic and Research Development

Multiple studies revealed mentorship’s critical role in fostering research engagement and scholarly productivity among surgical trainees. Torres et al. [17] reported that after implementing a structured research mentorship program, residents produced more publications per capita (1.15 vs. 0.79) and achieved a higher journal impact factor (1.25 vs. 0.55 per resident) compared to pre-program participants. Additionally, a greater proportion of residents pursued subspecialty fellowships following the program (81.5% vs. 45.8%), suggesting mentorship’s influence on continued academic engagement (Table 3). Similar findings were observed by Haas et al. [24], whose interdisciplinary resident research mentorship team (RRMT) led to increased publication and presentation rates (57.5% vs. 28.4% and 50% vs. 16.2%, respectively). The mentorship intervention independently predicted higher research productivity (odds ratio (OR) = 3.62, 95% confidence interval (CI) = 1.57-8.31), even when adjusting for fellowship pursuit.

Coyan et al. [19] demonstrated comparable results in cardiothoracic surgery residents. Their structured mentorship program, which incorporated biostatistical guidance and frequent mentor meetings, resulted in a marked increase in academic output, with publications rising from 0.6 ± 1 to 4.1 ± 5.6 per resident (p = 0.01) and presentations doubling post-intervention (1.1 to 2.1 per resident, p < 0.01). Baig et al. [20] further established that the mentor’s academic experience itself was a determinant of research success: resident projects supervised by mentors with more than 25 publications were significantly more likely to be published (OR = 2.46, p = 0.042). Collectively, these studies [17,19,20,24] affirm that structured and sustained mentorship fosters measurable academic advancement among surgical trainees (Table 1).

Clinical Competence and Skill Development

Several studies highlighted mentorship’s positive influence on clinical and technical skill acquisition. Schmalbach et al. [21] introduced a resident reviewer development program pairing trainees with seasoned reviewers. Mentees achieved a 94% pass rate on independent review testing, with scores exceeding journal averages, and 28% attained “Star Reviewer” recognition. Similarly, Aho et al. [22] found that mentor-guided self-directed learning (SDL) in laparoscopic training significantly improved all assessed skills (p < 0.05). Residents under mentorship reported higher practice engagement and self-assessed performance compared to peers. Schroll et al. [18] extended this finding into quality improvement (QI) education, where mentored residents demonstrated increased participation in QI projects (from 0.4 to 1.8 per resident) and improved confidence in developing and implementing QI initiatives. These findings indicate that mentorship enhances hands-on learning, self-reflection, and performance outcomes (Tables 3, 4).

Professional Identity and Leadership Formation

Mentorship also contributed to residents’ professional identity formation and leadership capacity. Ratan et al. [25] evaluated a mentorship-based leadership program within obstetrics and gynaecology. Although improvements in self-reported leadership scores did not reach statistical significance (3.82 to 3.96, p = 0.3), both mentors and mentees rated the experience highly (mean satisfaction of 4.62-5.29 on a six-point scale). Similarly, Haas et al. [24] and Mandel et al. [29] found mentorship influential in shaping academic and career trajectories. Mandel et al. [29] reported that residents in mentorship-enriched programs were significantly more likely to pursue academic careers (44% vs. 0%, p = 0.026) and maintain research involvement after graduation (79% vs. 0%, p < 0.001). This aligns with Khan et al.’s [28] findings in neurosurgery programs, where established mentorship structures (≥5 years) correlated with superior board pass rates (oral: 98% vs. 94%; written: 85% vs. 76%) and greater publication productivity (15.57 vs. 7.7, p < 0.005). These results highlight mentorship’s long-term influence on shaping surgical professionalism, leadership skills, and commitment to academic medicine (Table 1).

Psychosocial Support and Well-being

Beyond academic and technical outcomes, mentorship provided crucial psychosocial benefits. Shah et al.’s [27] systematic review across surgical subspecialties found mentorship to be a protective factor against resident burnout. Programs incorporating regular mentor-mentee interaction improved well-being, resilience, and work-life balance. Rajendran et al. [23] evaluated a peer mentorship initiative among general surgery residents and found that 77% of mentees reported the program as helpful across domains, including confidence, performance, and goal setting. Notably, 100% acknowledged it was at least slightly beneficial for goal establishment. Mentors themselves reported comparable benefits, underscoring the bidirectional value of mentorship. These findings reinforce mentorship’s role as both an academic and emotional support mechanism, crucial in mitigating stress and fostering belonging in high-demand training environments (Table 1).

Peer and Near-Peer Mentorship

Peer mentorship emerged as an accessible and effective complement to faculty-led models. Rajendran et al.’s [23] peer-pairing approach between PGY1 and PGY2 residents facilitated mutual support and skill-sharing, particularly during the transition into clinical training. Salles et al. [26] similarly implemented a junior-senior resident pairing system supported by quarterly social meetings. While engagement varied, only 23% met their mentors at least once, the majority of the participants who did engage rated the experience positively. These near-peer programs demonstrated potential for enhancing collegiality, communication, and mentorship continuity within hierarchical surgical teams (Table 3).

Challenges and Limitations of Mentorship Programs

Despite the evident benefits, several barriers to effective mentorship were identified (Table 2). Time constraints and heavy clinical workloads were the most frequently cited obstacles across studies [17,23,27]. Both mentors and mentees struggled to maintain regular meetings, leading to inconsistent engagement. Mentor availability and training also emerged as key limitations; few institutions provided structured mentor development programs, resulting in variable quality and preparedness. Schmalbach et al. [21] noted that mentor shortages limited cohort size and extended program duration, while Salles et al. [26] and Ratan et al. [25] found inconsistent participation due to scheduling conflicts and lack of accountability structures.

Program sustainability was another recurrent challenge. Informal or ad hoc mentorship models often led to unclear expectations and uneven outcomes [23,24]. Conversely, formalised mentorship frameworks with defined goals, regular assessments, and institutional recognition, such as the RRMT [24] and the research committee model [17], yielded more consistent results. Financial and administrative burdens also affected feasibility: Schmalbach et al.’s [21] program required four to six hours of weekly administrative support, while Haas et al. [24] reported annual departmental costs of approximately $46,000.

Finally, evaluation and outcome measurement were inconsistent. Most studies relied on self-reported satisfaction and subjective metrics rather than standardised assessment tools. Only a few, such as Haas et al. [24] and Khan et al. [28], incorporated objective measures such as publication rates or board examination results. This heterogeneity complicates cross-study comparisons and underscores the need for validated instruments to assess mentorship effectiveness in surgical education.

Discussion

Principal Findings

This review synthesised evidence from 13 studies examining the role of mentorship in postgraduate surgical education. Across diverse surgical specialties, mentorship consistently demonstrated positive effects on residents’ academic productivity, clinical competence, leadership development, and psychological well-being. Structured mentorship programs, particularly those integrated into research or educational frameworks, were associated with significant improvements in scholarly output and skill acquisition [17-19,22,24].

Mentorship was found to enhance residents’ engagement in research activities and QI initiatives, increase publication and presentation rates, and foster professional identity formation. For example, Torres et al. [17] and Haas et al. [24] demonstrated marked increases in publication rates and academic presentations following implementation of formal research mentorship programs, while Coyan et al. [19] reported a fourfold increase in publications per resident after introducing structured mentorship in cardiothoracic surgery. Similarly, Schroll et al. [18] observed greater involvement and confidence in QI projects when mentors provided ongoing feedback.

Mentorship also positively influenced psychosocial and career outcomes. Shah et al. [27] identified mentorship as a protective factor against burnout, while Rajendran et al. [23] and Salles et al. [26] demonstrated that peer and near-peer mentoring improved confidence, goal setting, and sense of belonging among residents. At the institutional level, programs with long-standing mentorship structures were linked with superior board pass rates and academic career progression [28,29].

However, persistent challenges were noted. Time constraints, insufficient mentor training, and lack of formal program structure limited the frequency and quality of mentor-mentee interactions [21,23,26]. Programs with protected time, administrative support, and institutional recognition achieved higher engagement and sustainability.

Comparison With Existing Literature

The findings from this review align with existing literature emphasising mentorship as a cornerstone of postgraduate medical and surgical education. Numerous reviews [30,31] have reported mentorship as one of the strongest predictors of academic success and career satisfaction among physicians. Consistent with the results of Haas et al. [24], Torres et al. [17], and Coyan et al. [19], previous work has shown that mentored trainees are more likely to publish research, present at conferences, and pursue academic careers [32,33].

Mentorship also reinforces professional identity formation, as demonstrated by Ratan et al. [25] and Mandel et al. [29], echoing [34], who described mentorship as essential for the socialisation of trainees into the values and roles of the surgical profession. Furthermore, mentorship enhances reflective practice and feedback, key components of competency-based medical education [35,36].

Peer mentorship findings [23,26] parallel recent studies in medical education highlighting the accessibility and relatability of near-peer mentors, which reduce hierarchical barriers and promote psychological safety [37,38]. However, this review also underscores mentorship’s dependence on contextual and institutional support. The variability in program outcomes observed among studies mirrors global findings that mentorship effectiveness is contingent upon structured frameworks, adequate mentor preparation, and formal evaluation mechanisms [39,40].

Overall, the evidence from this review extends the current understanding by quantitatively demonstrating mentorship’s impact across multiple outcome domains, particularly academic output and technical skill acquisition, while reinforcing its qualitative value in professional development and well-being.

Strengths and Limitations of the Evidence

The studies included in this review represent a broad cross-section of surgical disciplines and educational models, strengthening the generalisability of the findings. The inclusion of both quantitative outcomes (e.g., publication rates, board pass percentages, Likert-scale confidence measures) and qualitative assessments (e.g., perceived value, satisfaction) allows for a comprehensive understanding of mentorship’s multidimensional impact.

However, several methodological limitations were evident across the included studies. First, study design heterogeneity limits direct comparison: most studies were observational or cohort-based, with few employing randomisation or control groups. This introduces potential confounding factors, such as institutional culture, resident motivation, and concurrent educational interventions, that may have influenced outcomes.

Second, self-reported data were prevalent. Studies such as Salles et al. [26] and Ratan et al. [25] relied on resident perceptions rather than objective measures, increasing the risk of social desirability bias. Additionally, follow-up durations were often short, making it difficult to assess the long-term sustainability of mentorship effects. Torres et al. [17], for instance, found that the initial gains in publication output were not sustained post-residency.

Third, mentor-related factors, such as experience, commitment, and interpersonal skills, were inconsistently defined and measured. Although Baig et al. [20] demonstrated that mentor academic productivity correlated with mentee success, few studies evaluated how mentor training or feedback quality influenced outcomes.

Finally, evaluation frameworks were inconsistent. While Haas et al. [24] used objective academic measures and statistical analyses, others, such as Rajendran et al. [23], relied on descriptive or survey-based metrics. This lack of standardisation underscores the need for validated instruments to evaluate mentorship effectiveness within surgical education.

Implications for Practice and Future Research

The evidence supports the integration of structured mentorship programs as a core component of postgraduate surgical curricula. Institutions should prioritise the allocation of protected mentorship time within residency schedules, ensuring both mentors and mentees can engage meaningfully. Administrative and financial support, as exemplified by Haas et al. [24] and Schmalbach et al. [21], is essential for sustainability.

Faculty development should include formal mentor training in feedback delivery, educational theory, and cultural competence. Programs such as those described by Ratan et al. [25] and Rajendran et al. [23] demonstrate the potential of combining faculty and peer mentorship to address both academic and psychosocial needs. Furthermore, mentorship should be institutionally recognised as an academic contribution, with appropriate credit in promotion and appraisal systems.

Future research should aim to develop standardised outcome measures that capture both objective and subjective mentorship effects; conduct longitudinal studies assessing mentorship’s impact on career progression, leadership attainment, and retention in academia; explore the role of digital and group-based mentorship in addressing workforce and time limitations; and evaluate mentorship interventions across diverse populations, including underrepresented groups, to promote equity in surgical training.

## Conclusions

This review reaffirms mentorship as an indispensable element of postgraduate surgical education. It fosters research engagement, skill development, leadership, and well-being among trainees, while enhancing academic culture and institutional success. Structured and well-supported mentorship frameworks yield the most consistent benefits, but sustained commitment from institutions is vital to overcome barriers of time, training, and evaluation. As surgical education increasingly embraces competency-based and holistic assessment models, mentorship must remain central: not as an optional adjunct but as a foundational pillar in cultivating skilled, resilient, and compassionate surgical professionals.
